# Exploring the Mechanism of the Palladium-Catalyzed 3-Butene-2-ol Amination Reaction: A DFT Study

**DOI:** 10.3389/fchem.2020.00048

**Published:** 2020-02-21

**Authors:** Lingshan Lyu, Wei Feng, Siwei Yang, Huiling Liu, Xuri Huang

**Affiliations:** ^1^Laboratory of Theoretical and Computational Chemistry, Institute of Theoretical Chemistry, Jilin University, Changchun, China; ^2^College of Material and Chemical Engineering, Tongren University, Tongren, China

**Keywords:** amination, alkene alcohol, palladium catalyzed, axial chirality, DFT calculations

## Abstract

Palladium-catalyzed asymmetric allylic substitution, due to its valuable reactive profile, has become a quite useful tool in organic synthesis fields. In the present study, density functional theory (DFT) calculations were applied to investigate the important factors for palladium-catalyzed 3-butene-2-ol and methylaniline amination reaction, with tetrahydrofuran (THF) as solvent. We find that this catalytic protocol results in high regio- and stereoselectivity, which is in line with the experimental result. According to our calculations, the high regio- and stereoselectivity is caused by the steric hindrance between the substrate and the catalyst ligand. To verify this point, we further explore the reactive process with different axial chirality on the catalyst ligand (altering the steric hindrance), and the results suggest that the preponderant R chiral configuration product has reversed. These results could lead to a better understanding of the mechanism for 3-butene-2-ol amination reaction and are helpful for the design of the corresponding catalyst ligand in the industry.

## Introduction

The N-alkylation of amines is an important kind of organic synthetic reaction. The products of such reactions relate to many important fields such as defense, chemical, and pharmaceutical. As the development of chemical and catalytic synthesis technologies, the C–N bond formation reaction via N-alkylation has become one of the focused researches in the organic chemistry. The early studies about the C–N bond formation reaction includes the famous Ullmann reaction (Ullmann and Bielecki, 1901) and Goldberg reaction (Goldberg, 1906). Subjected to the experimental condition, these approaches generally form much halogen hydride and acid wastewater, which is harmful for the environment. The reactive conditions are also tough due to the absence of a suitable catalyst, and the atom utilization and the product selectivity are also unsatisfactory. The discovery of the Buchwald–Hartwig N-alkylation method (Hartwig et al., 1996, 1999; Fors and Buchwald, 2010) boosts the development of the C–N bond's establishment and introduces palladium catalyzed into the aryl halide N-alkylation reactions. However, this method still suffers environment-unfriendly issues.

With the development of the green chemistry, exploring the reactions with higher efficiency and less toxicity has been the goal for scientists. Alcohol has drawn more and more attention because it has equal characters with the aryl halide in N-alkylation reactions (Trost and Crawley, 2003; Lu and Ma, 2008; Zhang et al., 2014). This reactive profile has many advantages: the raw reactants are cheap to obtain, the yield and the selectivity are considerable, and the only by-product is water. In 2002, Ozawa and coworkers found that allylic alcohols can be used as coupling factors of allylic substitution reactions catalyzed by Pd (Ozawa et al., 2002). Then, they found that the C–O bond dissociation is the rate-determining step in these reactions (Ozawa et al., 2004), which was also demonstrated in le Floch's work using density functional theory (DFT) calculation approaches (Piechaczyk et al., 2006). Further research revealed a more detailed reaction process of the oxidative addition step (Kinoshita et al., 2004; Raducan et al., 2012; Sundararaju et al., 2012). In 2014, Banerjee and co-workers chose Pd(dba)_2_ as catalyst to catalyze the 2-cyclohexene-1-ol and aniline amination reaction; they tested and optimized a series of reactive conditions (including ligands, additive, solvent, and temperature). Finally, they found that the yield was nearly 100% and the *e.r*. was 96:4–85:15 with **L**_**8**_ as the ligand, and 298K and tetrahydrofuran (THF) as the solvent (Banerjee et al., 2014). They also determined that the chiral phosphoric acid in the catalytic ligand could promote the formation of the active π-(allyl)-Pd intermediate, even changing the 2-cyclohexene-1-ol to a low-active chain alkyl allyl alcohol; the results of the reaction were also ideal. More broadly effective palladium catalysts for nucleophiles have also been determined over the years (Yang et al., 2015, 2016; Jiang et al., 2017). Recently, it has been reported that the chirality of catalytic ligand could significantly influence the stereoselective coordination (Major et al., 2019). In consideration of the enormous raw reactants and catalysts mentioned above, the mechanism of the high enantioselectivity for the N-alkylation reaction still needs further elaboration. Exploring the reactive process *in silico* thus becomes helpful and necessary.

Up to now, many corresponding issues have been successfully solved by the DFT approach (Herz et al., 2019; Ramakrishnan et al., 2019; Ying et al., 2019). Thus, in the present study, we explored the N-alkylation reactive process between 3-butene-2-ol and the methylaniline detailedly using the DFT approach. With THF as solvent and different axial-chirality ligands (**L** and **L′**) on the catalyst, the different attack directions of the nucleophile were considered, and the energetic profiles of the reaction were obtained. At an atomic view, the results will demonstrate the preferred formation of the chiral product and reveal the cause of the high regio- and stereoselectivity.

## Methods

**Figure 1:**
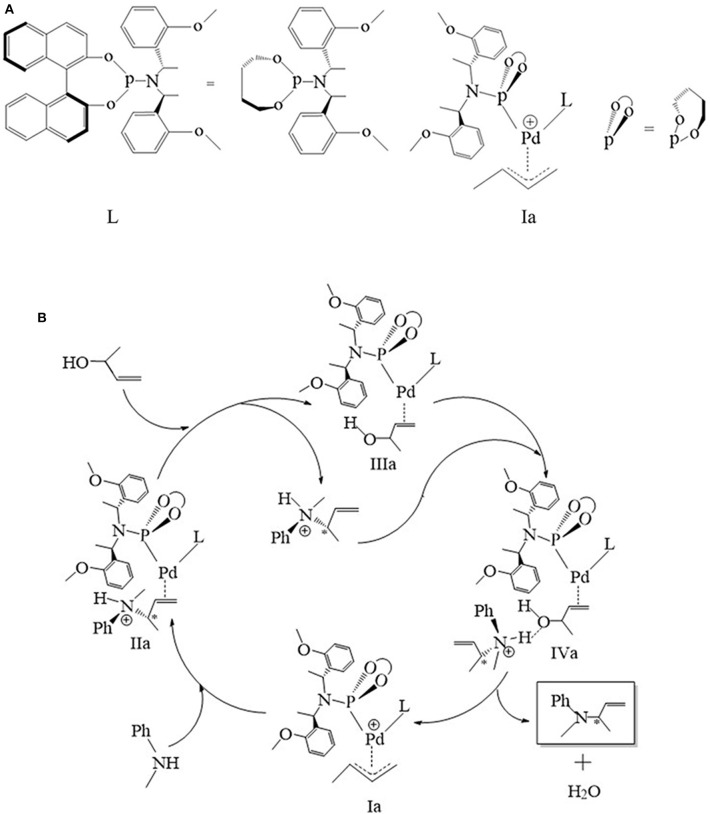
**(A)** The structure of phosphoramidite ligand (**L**). **(B)** The schematic representation of the mechanism of palladium-catalyzed the 3-butene-2-ol amination reaction.

All calculations based on density functional theory were performed using the Gaussian 09 (Frisch et al., 2009) (Rev D. 01) software package. The structures were fully optimized by BP86 functional (Perdew, 1986; Becke, 1988), with def2-TZVP basis set on the Pd atom (Schäfer et al., 1992) and the 6-31G (d) basis set on the C, H, O, N, and P atoms (Hehre et al., 1972; Hariharan and Pople, 1973). The polarizable continuum model (PCM) method was used and THF was chosen as the solvent, and Grimme's version 3 dispersion corrections (DFT-D3, disp3 version) (Grimme et al., 2010) were introduced. Besides, the Gibbs free energy with thermal correction was obtained from the frequency calculations, and the single-point energies were also performed on BP86 level with an all-atom def2-TZVP basis set (Weigend et al., 2003), empirical dispersion corrections (DFT-D3), and PCM method for THF solvent. Intrinsic reaction coordinate (IRC) calculations (Fukui, 1970, 1981) were performed to verify if the transition states coordinated with the intermediate correctly. The atomic dipole-corrected Hirshfeld (ADCH) (Lu and Chen, 2012b) was calculated by the Multiwfn 3.7 (Lu and Chen, 2012a) program. The bond length of the key step is shown in Schemes S1–S6 and Tables S1–S6, respectively (detailed in the supporting information, they are not shown in the manuscript for clarity). In addition, the configuration of the phosphoramidite ligand (**L**) is shown in Figure 1A. The ligand was simplified by removing the bulky backbone atoms of naphthalene and just remained the chiral character. The whole catalytic process is illustrated in Figure 1B, and the accurate configuration of each state are shown in the corresponding figures in each later section.

## Result and Discussion

### The 3-Butene-2-ol Amination Reaction Process in Gas

**Figure 2:**
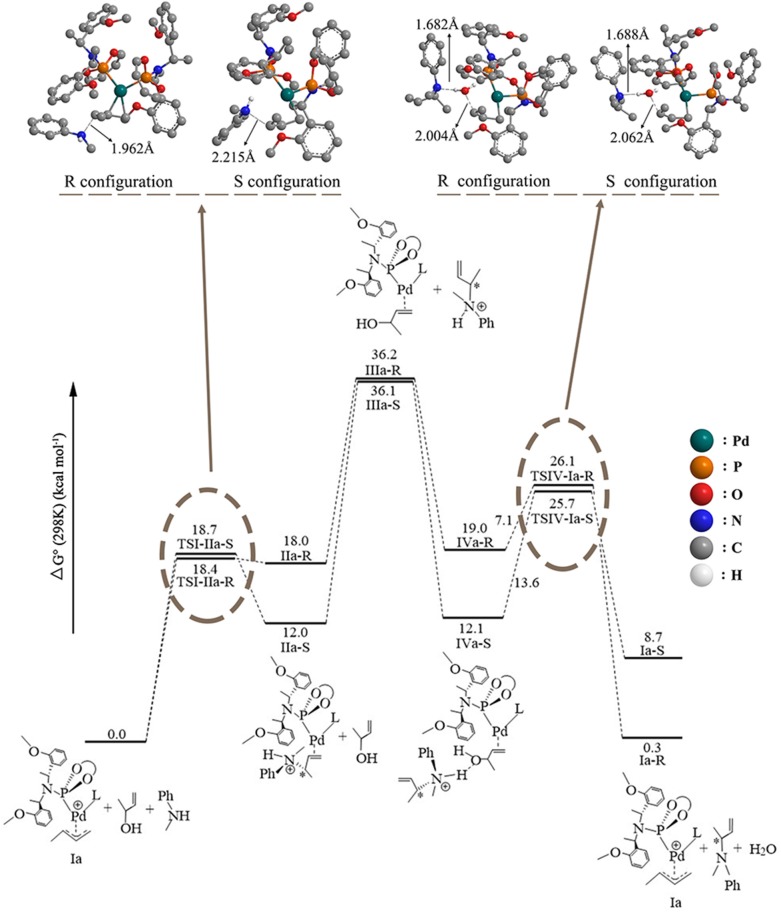
The overall free energetic profiles for the 3-butene-2-ol amination reaction process in gas catalyzed by phosphoramidite palladium.

**Figure 3:**
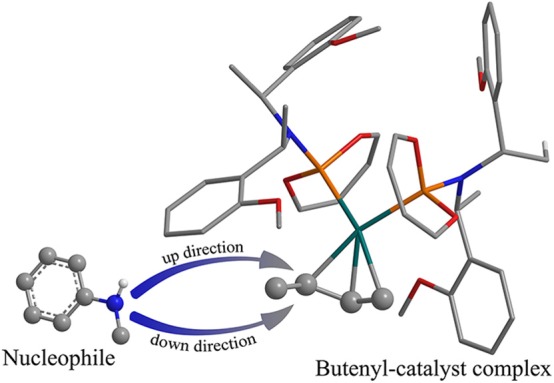
The two attack directions of the nucleophile to approach the substrate complex.

The overall free energetic profile for the 3-butene-2-ol amination reaction process in gas is outlined in Figure 2. The total energy of palladium-butenyl intermediate **Ia**, 3-butene-2-ol and methylaniline, was set to the energy reference. Butenyl, which coordinates with Pd of **Ia**, was identified as having a three-center–four-electron π bond, which causes it to lack electron, while the N of methylaniline has lone pair electrons and could act as the nucleophile. Thus, the methylaniline could nucleophilic attack the butenyl moiety of complex **Ia** in two directions (named as up-direction and down-direction, as shown in Figure 3), and form two transition states (see Figure 2). For the up-attack direction of the nucleophile, it could form the S chiral configuration transition state, **TSI-IIa-S** (18.7 kcal/mol); for the down-attack direction, it forms the R chiral configuration transition state, **TSI-IIa-R** (18.4 kcal/mol). The energy barrier of forming these two configuration transition states are very similar. The distance between the N of methylaniline and the C of the butenyl is 1.962 Å in **TSI-IIa-R** and 2.215 Å in **TSI-IIa-S**, while it decreases to 1.582 Å in **IIa-R** and 1.565 Å in **IIa-S** (Table S1), which suggests that the methylaniline forms the covalent bond with the butenyl. In **IIa-R/S**, the conjugative effect between the butenyl and Pd decreases as the covalent binding between the butenyl and the N of methylaniline increases, which causes the total electron transfers from N to the Pd. Then, the 3-butene-2-ol participates in the reaction, and its butenyl moiety coordinates with the palladium and replaces the *N*-butenyl-*N*-methylaniline, forming **IIIa-R** (36.2 kcal/mol) and **IIIa-S** (36.1 kcal/mol). It seems that the energy value of these two states are a little high. The abnormal energy values indicate that the reactive process is not so satisfactory in gas condition; therefore, the influence of the solvation effect on the reaction is considered for further exploration.

### The 3-Butene-2-ol Amination Reaction Process in THF

**Figure 4:**
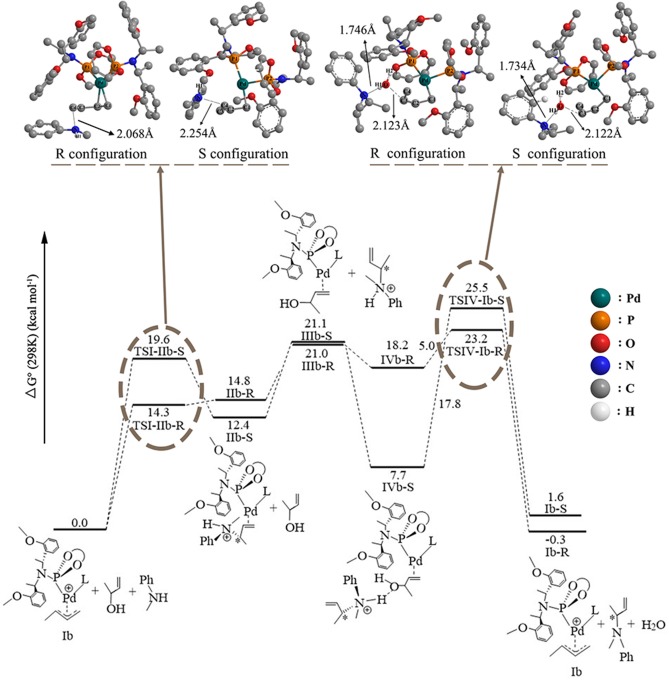
The overall free energetic profiles for the 3-butene-2-ol amination reaction process in THF catalyzed by phosphoramidite **Pd-L**. The structure of the **TSI-IIb-R/S** and **TSIV-Ib-R/S** has been shown in ball-and-stick style; the important distance between atoms has also been shown in the corresponding place.

**Figure 5:**
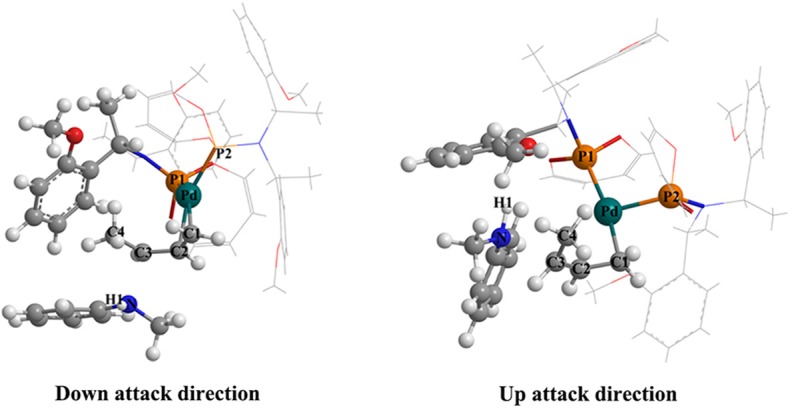
Visualizing the structure of the **TSI-IIb-R** state (left picture) and the **TSI-IIb-S** state (right picture).

To consider the effect of the solvent for the reaction, THF was chosen as the representative solvent in our work because it was proved to be the best solvent for the reaction (Banerjee et al., 2014). The overall free energetic profile is shown in Figure 4. The butenyl that coordinates with Pd of **Ib** was found having a three-center–four-electron π bond, and the electron density is unsaturated at this portion, while the N of methylaniline has lone pair electrons and could act as the nucleophilic reactive site. There are two nucleophilic attack directions for N, which could form the R and S configuration transition states, respectively. The distance between the N of methylaniline and the C of butenyl is 2.086 Å in **TSI-IIb-R** and 2.254 Å in **TSI-IIb-S**, while it decreases to 1.575 Å in **IIb-R** and 1.559 Å in **IIb-S** (Table S3), which suggests that the methylaniline forms the covalent bond with the butenyl. For the up-attack direction of the nucleophile, it could form the S chiral configuration transition state, **TSI-IIb-S** (19.6 kcal/mol); for the down-attack direction, it forms the R chiral configuration transition state, **TSI-IIb-R** (14.3 kcal/mol). Compared with the corresponding energy values in gas, **TSI-IIa-S** (18.7 kcal/mol) and **TSI-IIa-R** (18.4 kcal/mol), the influence of solvent environment to the reaction has been demonstrated clearly. The energy barrier of forming the R configuration is lower than the S configuration, which means that it is easier for the nucleophile to attack the butenyl form the down direction. To figure out the difference in the energy barrier, we visualized the structure of the **TSI-IIb-R** state and the **TSI-IIb-S** state. As shown in Figure 5, it could be seen that, for the down-attack direction, the methylaniline locates just below the butenyl. Thus, it can coordinate with the butenyl directly. However, for the up-attack direction, the methyl of the methylaniline forms steric hindrance with the phosphoramidite ligand (**L**), which makes methylaniline combining difficult and enlarges the energy barrier. In the next step, the 3-butene-2-ol participates in the reaction, and its butenyl moiety coordinates with the palladium, replacing the *N*-butenyl-*N*-methylaniline, and forms **IIIb-R** (21.0 kcal/mol) and **IIIb-S** (21.1 kcal/mol). The energy value of the **IIIb-R** state and the **IIIb-S** state becomes distinctly lower than that in gas (36.2 and 36.1 kcal/mol), which may be related to the existence of the THF. Thus, we can suggest that the existence of the THF increases the strength of the coordination bond, which would be described at the charge analysis part. The dissociative cationic *N*-butenyl-*N*-methylaniline could further form a hydrogen bond with the substrate hydroxy of **IIIb-R** and **IIIb-S**, producing a relatively stable state **IVb-R** (18.2 kcal/mol) and **IVb-S** (7.7 kcal/mol).

**Figure 6:**
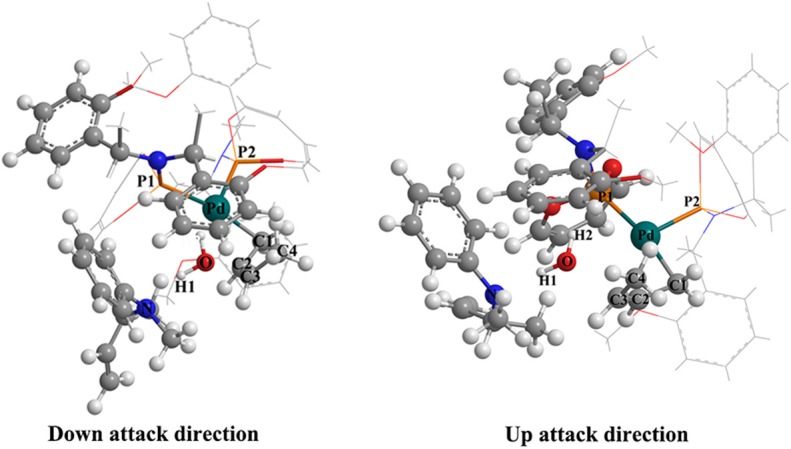
Visualizing the structure of the **TSIV-Ib-R** state (left picture) and the **TSIV-Ib-S** state (right picture).

The last step of the catalytic reaction contains the oxidative addition of the C–O bond in 3-butene-2-ol substrate, which produces water and deprotonated *N*-butenyl-*N*-methylaniline and regenerates the palladium-butenyl intermediate **Ib**. In the **IVb-R/S** state, the distance between the C and O of the 3-butene-2-ol is 1.467 Å/1.459 Å, and the distance between the N and H of the *N*-butenyl-*N*-methylaniline moiety is 1.073 Å/1.075 Å (Table S4). While in the **TSIV-Ib-R/S** state, the distance increases to 2.123 Å/2.122 Å and 1.746 Å/1.734 Å, which shows the elimination trend of the water molecule. The energy value of the **IVb-S** state (7.7 kcal/mol) is lower than the **IVb-R** state (18.2 kcal/mol), which seems that the **IVb-S** state is more stable. The energy barrier of forming the **TSIV-Ib-R** state is 5.0 kcal/mol, much lower than that of forming the **TSIV-Ib-S** state (17.8 kcal/mol). Besides, the energy value of the **TSIV-Ib-S** state is kind of high (25.5 kcal/mol), which causes it hard to be formed. To find the reason why the energetic barrier of the two states is so different, we visualized the structure of the **TSIV-Ib-R** and **TSIV-Ib-S** states (shown in Figure 6); it could be seen that, for the down-attack direction, the benzene ring of the *N*-butenyl-*N*-methylaniline locates just between the two benzene of the ligand, which is favorable for the reaction to proceed. However, for the up-attack direction, the benzene ring of the *N*-butenyl-*N*-methylaniline is close to the benzene ring of the ligand, which would form a strong steric hindrance and significantly enlarge the energy barrier. What is more, the final energy value of the **Ib-R** state is −0.3 kcal/mol, suggesting it is a slight exothermic reactive process, while the final energy value of the **Ib-S** state is 1.6 kcal/mol, suggesting it is a slight endothermic reactive process. These results identify that the down-attack direction of the nucleophile is the preferred reactive route. Combining the energy barrier values of the **TSI-IIb-R/S**, we can draw the conclusion: it is much easier to form R rather than S configuration product, under the **Pd-L** catalytic system condition with THF solvent. These results are in line with the experimental results (Banerjee et al., 2014) and could well-explain the highly regio- and stereoselective of the present catalytic protocol.

To better describe the reactive process of each step, we also calculate the ADCH population charge (Lu and Chen, 2012b) for the N (nucleophile) and the Pd (Table 1), owing to the main charge transformation taking place in these two atoms. In the beginning, due to the existence of the lone pair electrons, the ADCH charge for N is −0.406, an obvious negative value. The ADCH charge value for the Pd is 0.276, a positive value, because of its conjugation with the butenyl. After the nucleophilic attack of the nucleophile, in the **IIb-R/S** state, the methylaniline forms covalent bond with the butenyl, and the N shares electrons with the butenyl, which causes the ADCH charge of N increasing to 0.050/0.081 (**IIb-R/S**). The ADCH charge for the Pd decreases to 0.174/0.157 (**IIb-R/S**) as butenyl tends to form the conjugation with the nucleophile, which means that the electrons flow to the Pd. In the **IIIb-R/S** state, the ADCH charge for N and Pd basically maintains their value. However, due to the obvious energy difference existing in the **IIIa-R/S** and **IIIb-R/S** states, we also calculate the ADCH charge for the 3-butene-2-ol of the **IIIa-R/S** and **IIIb-R/S** states, and the value is −0.177 and −0.197, respectively. The more negative the ADCH charge is, the more stable the bond strength would be, since the total charge of the complex is electrically neutral. Therefore, the coordination bond between the 3-butene-2-ol and the Pd of the **IIIb-R/S** state is more stable than that of the **IIIa-R/S** state, which is in agreement with the existence of the THF increasing the strength of the coordination bond. In the **IVb-R/S** state, the ADCH charge for N is 0.072/0.050, and for Pd, ADCH charge is 0.108/0.147, respectively. The whole flow direction of the charge indicates that the down-attack direction of the methylaniline is the preferred reactive route in the Pd-L catalytic system, which enriches the reactive details.

**Table 1:** The ADCH charge of the N and the Pd in different states for **Pd-L** catalytic system.

**Stateatom**	**Methylaniline**	**Ib**	**IIb-S**	**IIb-R**	**IVb-S**	**IVb-R**
**N**	−0.406	–	0.081	0.050	0.050	0.072
**Pd**	–	0.276	0.157	0.174	0.147	0.108

### The Influence of the Ligand Axial Chirality on the Reaction

**Figure 7:**
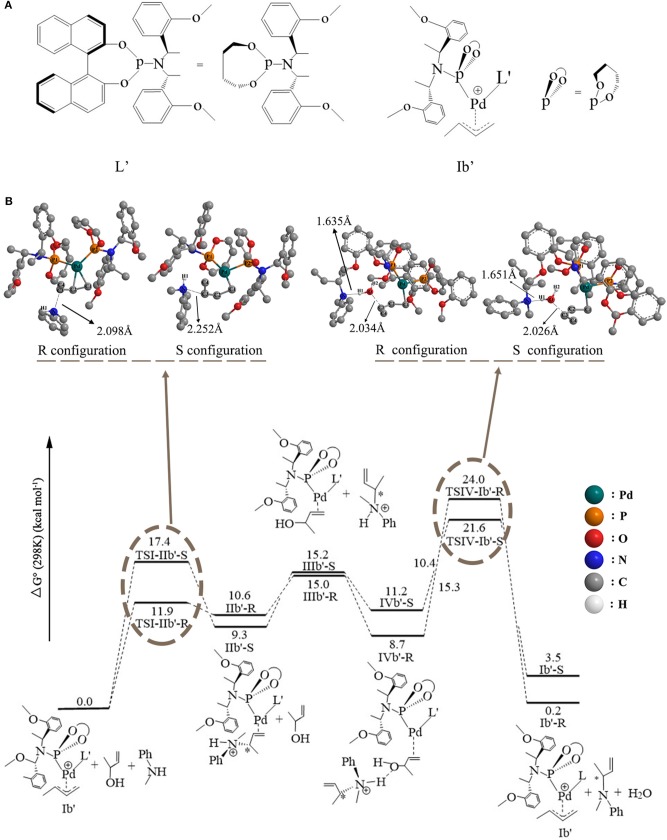
**(A)** The structure of phosphoramidite ligand (**L′**). **(B)** The overall free energetic profiles and structures for the 3-butene-2-ol amination reaction process in THF catalyzed by phosphoramidite **Pd-L′**.

To explore the influence of the ligand axial chirality on the reaction, the phosphoramidite ligand with different axial chirality of **L**, **L′** (Figure 7A, **L′** was also treated by the same way as **L**) was introduced. The whole free energetic profile has been shown in Figure 7B. The overall reactive mode is similar to that of the **Pd-L** catalytic system: For the up-attack direction of nucleophile, it could form the S chiral configuration, **TSI-IIb′-S** (17.4 kcal/mol); for the down-attack direction, it forms the R chiral configuration, **TSI-IIb′-R** (11.9 kcal/mol). Compared to the corresponding energy values of the **Pd-L** catalytic system, the **TSI-IIb-S** state (19.6 kcal/mol) and the **TSI-IIb-R** state (14.3 kcal/mol), the energy barrier of forming these two states becomes lower. The energy value of the **IIb′-R/S** state is 10.6 and 9.3 kcal/mol, respectively, which is also lower than the corresponding values of the **IIb-R/S** state (14.8 and 12.4 kcal/mol). That is to say, the **Pd-L′** catalytic system shows a better reactive ability to some extent. The distance between the N of methylaniline and the C of butenyl is 2.098 Å in the **TSI-IIb′-R** state and 2.252 Å in the **TSI-IIb′-S** state, while it decreases to 1.627 Å in the **IIb′-R** state and 1.556 Å in the **IIb′-S** state (Table S5), which is consistent with the covalent bonding trend between the methylaniline and the butenyl. Next, 3-butene-2-ol participates in the reaction, and its butenyl moiety coordinates with palladium, replacing the *N*-butenyl-*N*-methylaniline, forming **IIIb′-R** (15.0 kcal/mol) and **IIIb′-S** (15.2 kcal/mol). The dissociative cationic *N*-butenyl-*N*-methylaniline could further form a hydrogen bond with the substrate hydroxy of **IIIb′-R** and **IIIb′-S**, producing a relative stable-state **IVb′-R** (11.2 kcal/mol) and **IVb′-S** (8.7 kcal/mol). From Figure 7, we can see that the energy barriers of forming the **TSIV-Ib′-R** and **TSIV-Ib′-S** states are 15.3 and 10.4 kcal/mol, respectively. Based on the above results, the C–O bond dissociation is the rate-determining step for the reaction, which has also been proven by the experiment (Ozawa et al., 2004). Therefore, for the **Pd-L′** catalytic system, it is the S configuration product that seems easier to be formed, and this result is in contrast with the **Pd-L** catalytic system.

**Figure 8:**
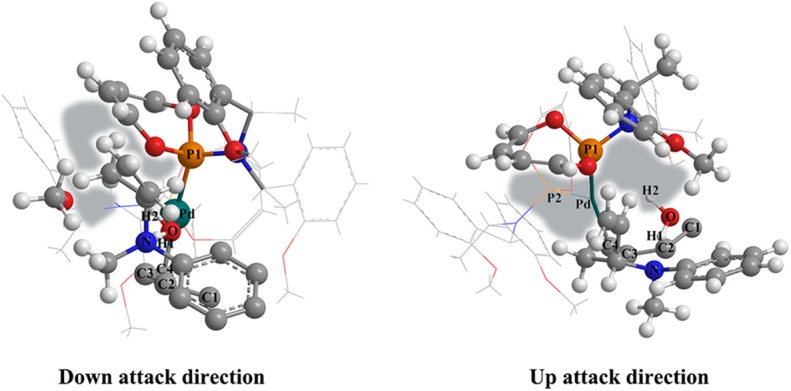
Visualizing the structure of the **TSIV-Ib′-R** state (left picture) and the **TSIV-Ib′-S** state (right picture). The space around the butenyl of the *N*-butenyl-*N*-methylaniline has been painted in light gray.

To figure out the reason why the ligand axial chirality could significantly influence the preponderant reactive route, we visualized the corresponding structure of the **TSIV-Ib′-R** and the **TSIV-Ib′-S** state. As shown in Figure 8, for the **TSIV-Ib′-R** state, the space around the butenyl of the *N*-butenyl-*N*-methylaniline is relative smaller than that of the **TSIV-Ib′-S** state. The relative small space could lead to a stronger steric hindrance in the R chiral configuration structure, which corresponds to the energy barriers of **TSIV-Ib′-S** (10.4 kcal/mol) and **TSIV-Ib′-R** (15.3 kcal/mol). Therefore, the axial chiral ligand greatly affected the preponderant reactive route by the steric hindrance. What is more, most energy values of the **Pd-L′** catalytic system are lower than that of the **Pd-L** catalytic system, suggesting that the **Pd-L′** catalytic system might be superior to the **Pd-L** catalytic system.

We also calculate the ADCH charge for the N (methylaniline) and the Pd (Table 2), which is similar to that of the **Pd-L** catalytic system process; the ADCH charge for N is −0.406, and the ADCH charge value for the Pd is 0.275. After the nucleophilic attack of the methylaniline, there is a covalent bond formed between the N and the butenyl. In the **IIb′-R/S** state, the ADCH charge of N increases to 0.016/0.070. In addition, because the butenyl tends to form the conjugation with the methylaniline, the electrons flow back to the Pd from the butenyl, which leads to the ADCH charge for the Pd decreasing to 0.085/0.086 (**IIb′-R/S**). In the **IIIb′-R/S** state, the ADCH charge for N and Pd basically maintains their value. The ADCH charge for the N is 0.070/0.082 in the **IVb′-R/S** state. The ADCH charge for the Pd is 0.159/0.110 in the **IVb′-R/S** state. These changes of the ADCH charge values also indicate that the up-attack direction of the methylaniline is the preferred reactive route in the Pd-L′ catalytic system and could help us to better understand the reactive mechanism.

**Table 2:** The ADCH charge of the N and the Pd in different states for **Pd-L′** catalytic system.

**Stateatom**	**Methylaniline**	**Ib′**	**IIb′-S**	**IIb′-R**	**IVb′-S**	**IVb′-R**
**N**	−0.406	–	0.070	0.016	0.082	0.070
**Pd**	–	0.275	0.086	0.085	0.110	0.159

## Conclusion

As the palladium-catalyzed asymmetry unsaturated olefin substitution reactions become more and more popular, understanding the catalytic mechanism has been an essential mission. In the present study, we utilized the DFT approach to explore the mechanism of palladium-catalyzed 3-butene-2-ol and methylaniline amination reaction. According to our computational results, the high regio- and stereoselectivity of the catalyst could influence the reactive route of the reaction, which induces different preponderant chiral product. This view could be proved by the high ratio of the chiral products in the experiment (Kimura et al., 2003; Piechaczyk et al., 2006; Banerjee et al., 2014). The high regio- and stereoselectivity is caused by the steric hindrance between the substrate and the ligand. Furthermore, this point is verified by introducing the axial chirality on the catalyst ligand. In the **Pd-L** catalytic system, down-attack direction of the nucleophile acts as the preponderant reaction route, during which process it tends to form R configuration product; in contrast, the up-attack direction of the nucleophile becomes the preponderant reaction route, and it tends to form S configuration product in the **Pd-L′** catalytic system. These results shed light on the formation process of the 3-butene-2-ol and methylaniline amination reaction, which might lead a deeper understanding between the catalyst ligand and the substrate and provide theoretical instructions for the catalyst ligand design and optimization in industry.

## Data Availability Statement

The raw data supporting the conclusions of this article will be made available by the authors, without undue reservation, to any qualified researcher.

## Author Contributions

LL performed research and wrote the article. WF and SY analyzed the data. HL and XH designed the research and wrote the article.

### Conflict of Interest

The authors declare that the research was conducted in the absence of any commercial or financial relationships that could be construed as a potential conflict of interest.
