# Supplementary material for: Exploring the Mechanism of the Palladium-Catalyzed 3-Butene-2-ol Amination Reaction: A DFT Study
Source: Front Chem. 2020 Feb 21;8:48. doi: 10.3389/fchem.2020.00048 (PMC7047136; doi:10.3389/fchem.2020.00048)
Supplement: Supplementary file 1 [file Data_Sheet_1.DOCX]

**Supporting Information**

**A DFT study on the palladium catalyzed butene alcohols amination reaction**

Lingshan Lyu^1^, Wei Feng^2^, Hanwen Chang^1^, Huiling Liu^1^*, Xuri Huang^1^*

^1^Laboratory of Theoretical and Computational Chemistry, Institute of Theoretical Chemistry, Jilin University, Changchun 130023, China

^2^College of Material and Chemical Engineering, Tongren University, Tongren, 554300, Guizhou, China

*** Correspondence:**Huiling Liu and Xuri Huang
huiling@jlu.edu.cn; huangxr@jlu.edu.cn

1. Bond Length

**
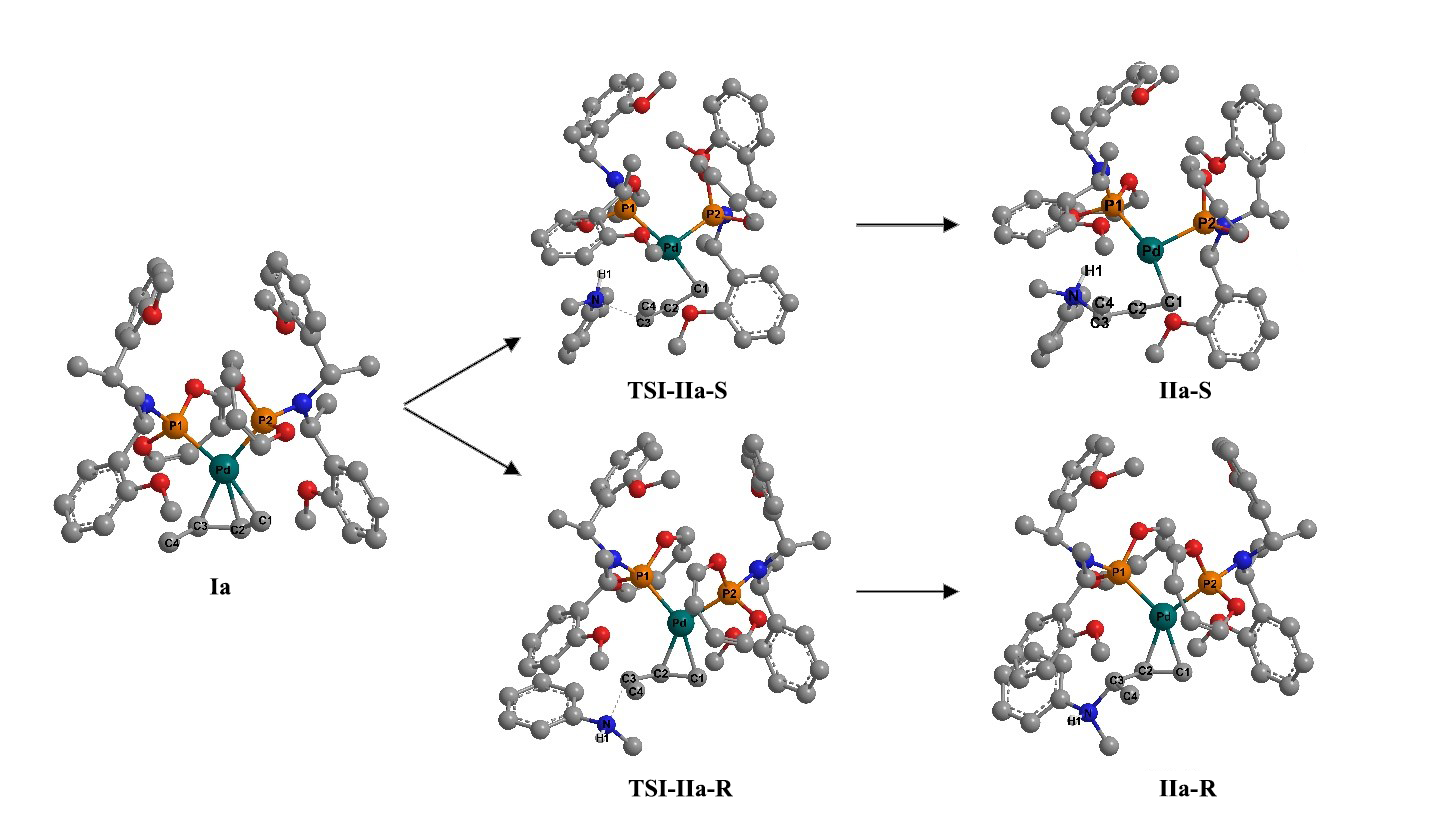
**

**Scheme S1.** In all structures the related atoms are labeled and the atom color is insistent with the manuscript.

**Table S1.** Bond lengths (Å) of the calculated structures of intermediates involved in nucleophilic attack.

| Bond | Ia | TSI-IIa-S | TSI-IIa-R | IIa-S | IIa-R |
| --- | --- | --- | --- | --- | --- |
| dPd-P1 | 2.295 | 2.323 | 2.287 | 2.277 | 2.291 |
| dPd-P2 | 2.290 | 2.271 | 2.276 | 2.306 | 2.284 |
| dPd-C1 | 2.212 | 2.103 | 2.134 | 2.210 | 2.142 |
| dPd-C2 | 2.172 | 2.388 | 2.137 | 2.278 | 2.142 |
| dPd-C3 | 2.247 | 3.150 | 2.855 | 3.176 | 2.998 |
| dPd-C4 | 3.088 | 3.609 | 3.409 | 3.588 | 3.638 |
| dC3-N | - | 2.215 | 1.962 | 1.565 | 1.582 |
| dN-H1 | - | 1.028 | 1.029 | 1.058 | 1.038 |

**
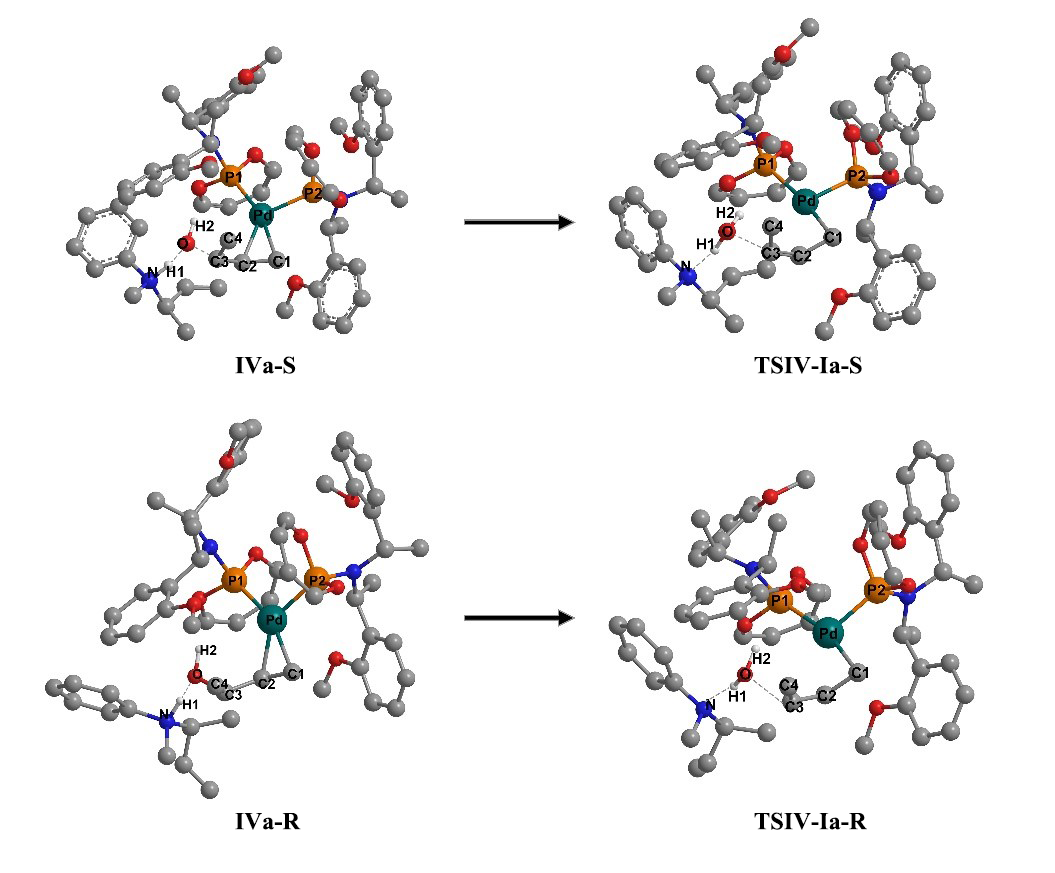
**

**Scheme S2.** In all structures the related atoms are labeled and the atom color is insistent with the manuscript.

**Table S2.** Bond lengths (Å) of the calculated structures of intermediates involved in oxidative addition step.

| Bond | IVa-S | IVa-R | TSIV-Ia-S | TSIV-Ia-R |
| --- | --- | --- | --- | --- |
| dPd-P1 | 2.282 | 2.262 | 2.335 | 2.326 |
| dPd-P2 | 2.303 | 2.290 | 2.271 | 2.275 |
| dPd-C1 | 2.183 | 2.146 | 2.107 | 2.119 |
| dPd-C2 | 2.250 | 2.172 | 2.376 | 2.320 |
| dPd-C3 | 3.209 | 2.158 | 3.131 | 3.158 |
| dPd-C4 | 3.647 | 3.610 | 3.648 | 3.816 |
| dC3-O | 1.466 | 1.473 | 2.062 | 2.004 |
| dO-H1 | 1.630 | 1.614 | 1.044 | 1.044 |
| dO-H2 | 0.992 | 0.992 | 0.991 | 0.989 |
| dN-H1 | 1.085 | 1.085 | 1.688 | 1.682 |

**
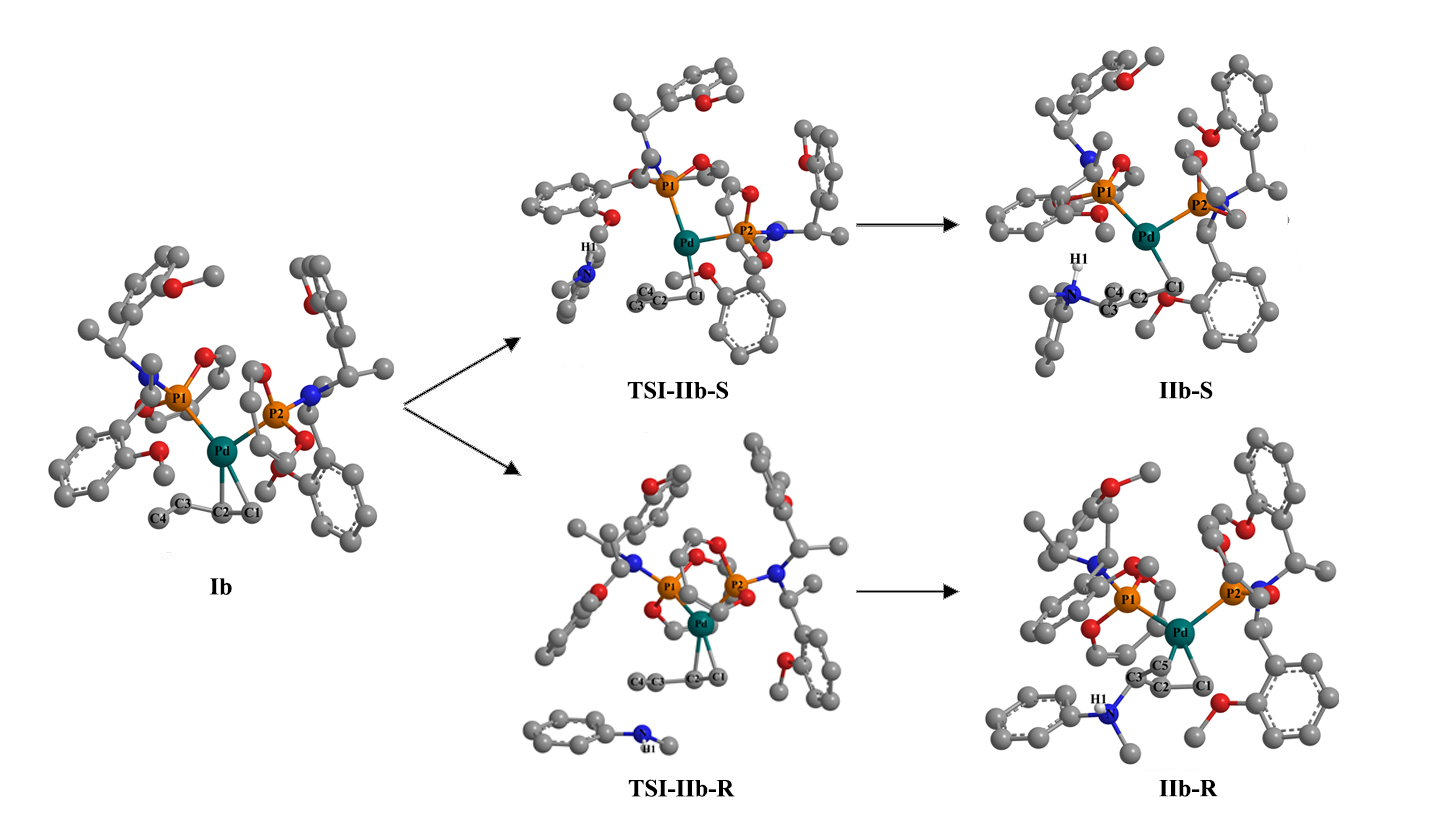
**

**Scheme S3.** In all structures the related atoms are labeled and the atom color is insistent with the manuscript.

| Bond | Ib | TSI-IIb-S | TSI-IIb-R | IIb-S | IIb-R |
| --- | --- | --- | --- | --- | --- |
| dPd-P1 | 2.296 | 2.337 | 2.287 | 2.293 | 2.288 |
| dPd-P2 | 2.294 | 2.279 | 2.266 | 2.305 | 2.282 |
| dPd-C1 | 2.212 | 2.090 | 2.159 | 2.187 | 2.149 |
| dPd-C2 | 2.178 | 2.466 | 2.157 | 2.282 | 2.148 |
| dPd-C3 | 2.258 | 3.304 | 2.793 | 3.216 | 3.040 |
| dPd-C4 | 3.071 | 3.648 | 3.339 | 3.599 | 3.677 |
| dC3-N | - | 2.254 | 2.068 | 1.559 | 1.575 |
| dN-H1 | - | 1.029 | 1.028 | 1.051 | 1.036 |

**Table S3.** Bond lengths (Å) of the calculated structures of intermediates involved in nucleophilic attack.

**
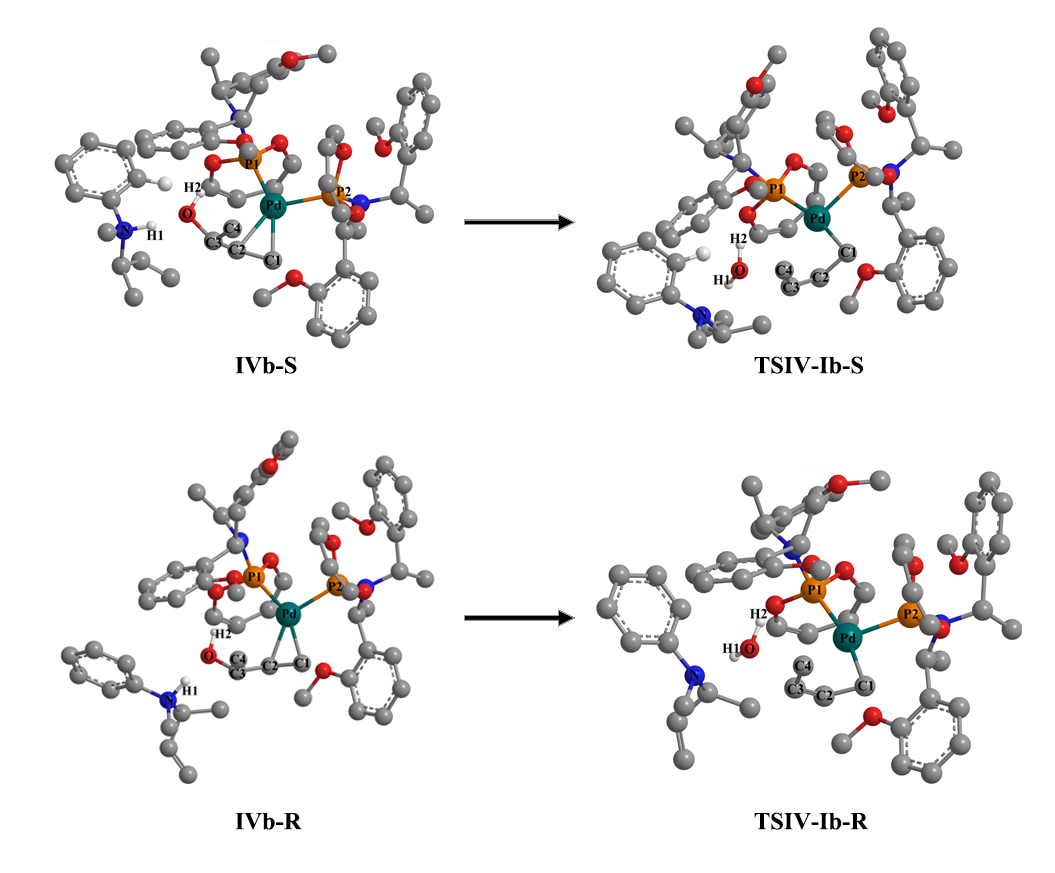
**

**Scheme S4.** In all structures the related atoms are labeled and the atom color is insistent with the manuscript.

**Table S4.** Bond lengths (Å) of the calculated structures of intermediates involved in oxidative addition step.

| Bond | IVb-S | IVb-R | TSIV-Ib-S | TSIV-Ib-R |
| --- | --- | --- | --- | --- |
| dPd-P1 | 2.270 | 2.266 | 2.326 | 2.334 |
| dPd-P2 | 2.292 | 2.287 | 2.266 | 2.268 |
| dPd-C1 | 2.186 | 2.158 | 2.093 | 2.095 |
| dPd-C2 | 2.248 | 2.187 | 2.363 | 2.359 |
| dPd-C3 | 3.193 | 3.182 | 3.193 | 3.158 |
| dPd-C4 | 3.575 | 3.610 | 3.763 | 3.684 |
| dC3-O | 1.459 | 1.467 | 2.122 | 2.123 |
| dO-H1 | 1.640 | 1.666 | 1.021 | 1.018 |
| dO-H2 | 0.989 | 0.989 | 0.984 | 0.986 |
| dN-H1 | 1.075 | 1.073 | 1.734 | 1.746 |


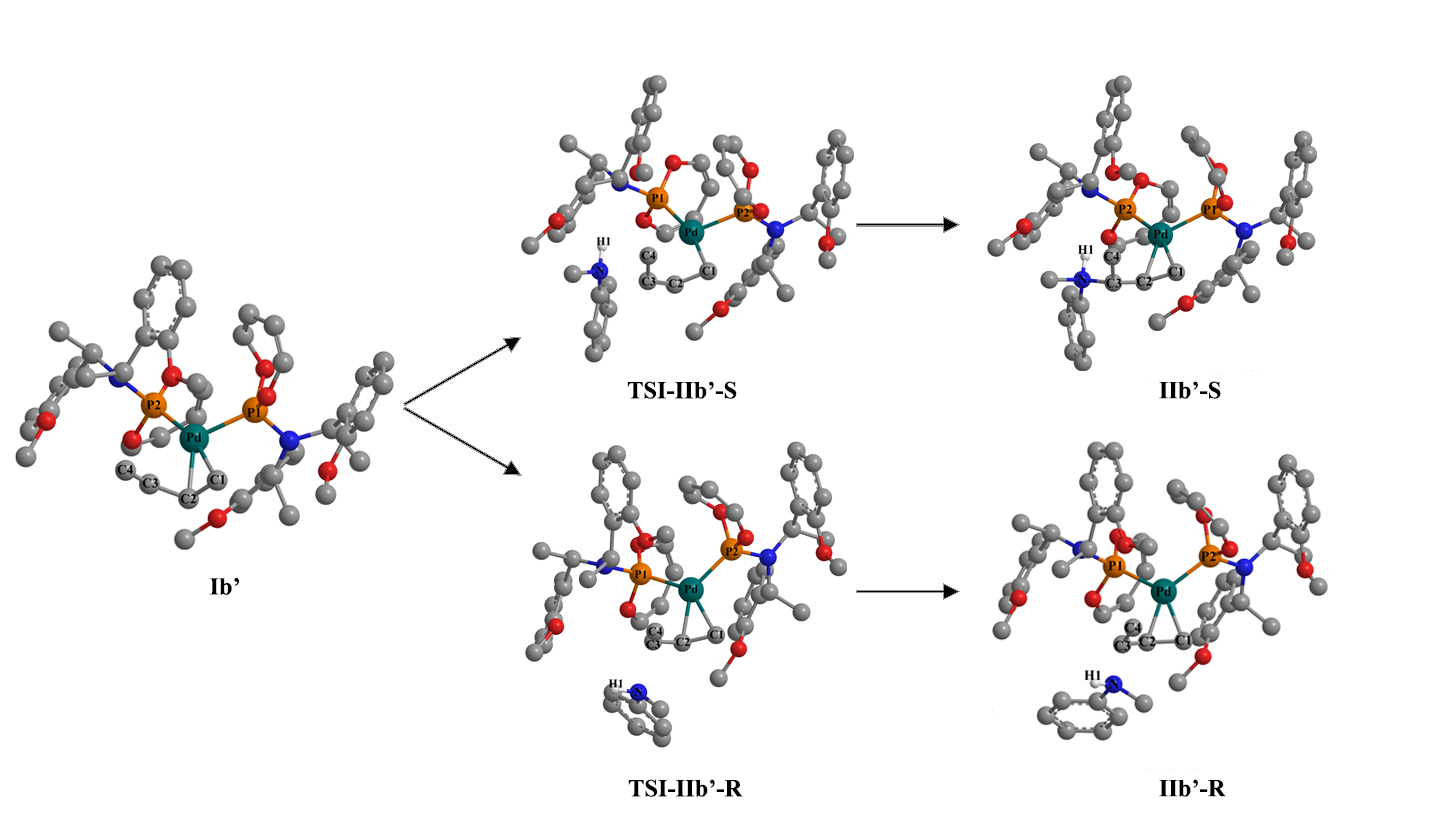


**Scheme S5.** In all structures the related atoms are labeled and the atom color is insistent with the manuscript.

**Table S5.** Bond lengths (Å) of the calculated structures of intermediates involved in nucleophilic attack.

| Bond | Ib’ | TSI-IIb’-S | TSI-IIb’-R | IIb’-S | IIb’-R |
| --- | --- | --- | --- | --- | --- |
| dPd-P1 | 2.287 | 2.301 | 2.303 | 2.274 | 2.291 |
| dPd-P2 | 2.301 | 2.274 | 2.279 | 2.309 | 2.282 |
| dPd-C1 | 2.216 | 2.095 | 2.186 | 2.153 | 2.166 |
| dPd-C2 | 2.175 | 2.407 | 2.153 | 2.199 | 2.141 |
| dPd-C3 | 2.264 | 3.248 | 2.820 | 3.242 | 3.088 |
| dPd-C4 | 3.143 | 3.653 | 3.463 | 3.614 | 3.496 |
| dC3-N | - | 2.252 | 2.098 | 1.556 | 1.627 |
| dN-H1 | - | 1.028 | 1.027 | 1.044 | 1.034 |


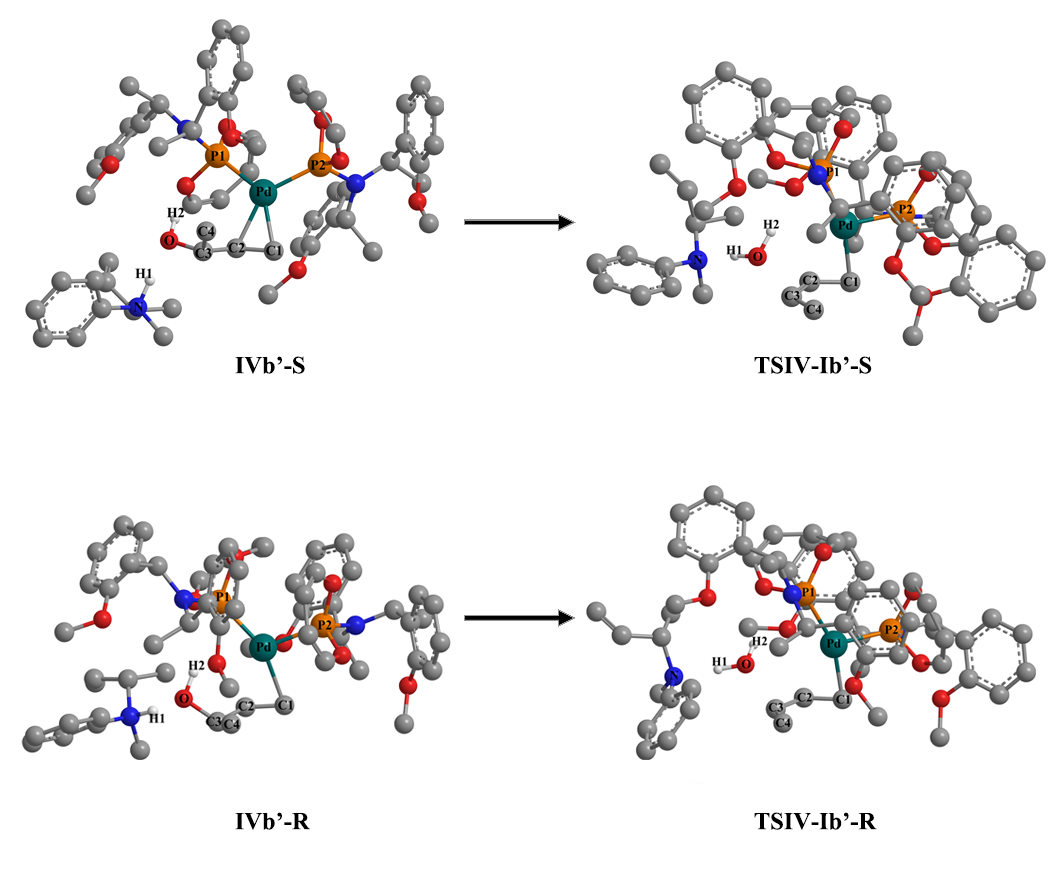


| Bond | IVb’-S | IVb’-R | TSIV-Ib’-S | TSIV-Ib’-R |
| --- | --- | --- | --- | --- |
| dPd-P1 | 2.275 | 2.261 | 2.294 | 2.311 |
| dPd-P2 | 2.288 | 2.315 | 2.268 | 2.267 |
| dPd-C1 | 2.217 | 2.211 | 2.115 | 2.117 |
| dPd-C2 | 2.225 | 2.267 | 2.363 | 2.383 |
| dPd-C3 | 3.232 | 3.160 | 3.195 | 3.172 |
| dPd-C4 | 3.517 | 3.570 | 3.706 | 3.719 |
| dC3-O | 1.467 | 1.469 | 2.026 | 2.034 |
| dO-H1 | 1.721 | 1.655 | 1.052 | 1.047 |
| dO-H2 | 0.997 | 0.996 | 0.989 | 0.987 |
| dN-H1 | 1.073 | 1.072 | 1.651 | 1.635 |

**Scheme S6.** In all structures the related atoms are labeled and the atom color is insistent with the manuscript.

**Table S6.** Bond lengths (Å) of the calculated structures of intermediates involved in oxidative addition step.
